# Arrays of individually controlled ions suitable for two-dimensional quantum simulations

**DOI:** 10.1038/ncomms11839

**Published:** 2016-06-13

**Authors:** Manuel Mielenz, Henning Kalis, Matthias Wittemer, Frederick Hakelberg, Ulrich Warring, Roman Schmied, Matthew Blain, Peter Maunz, David L. Moehring, Dietrich Leibfried, Tobias Schaetz

**Affiliations:** 1 Albert-Ludwigs-Universität Freiburg, Physikalisches Institut, Hermann-Herder-Strasse 3, Freiburg 79104, Germany; 2 Department of Physics, University of Basel, Klingelbergstrasse 82, Basel 4056, Switzerland; 3 Sandia National Laboratories, PO Box 5800 Albuquerque, New Mexico 87185-1082, USA; 4 Time and Frequency Division, National Institute of Standards and Technology, 325 Broadway, Boulder, Colorado 80305, USA; 5 Albert-Ludwigs-Universität Freiburg, Freiburg Institute for Advanced Studies, Albertstr. 19, 79104 Freiburg, Germany

## Abstract

A precisely controlled quantum system may reveal a fundamental understanding of another, less accessible system of interest. A universal quantum computer is currently out of reach, but an analogue quantum simulator that makes relevant observables, interactions and states of a quantum model accessible could permit insight into complex dynamics. Several platforms have been suggested and proof-of-principle experiments have been conducted. Here, we operate two-dimensional arrays of three trapped ions in individually controlled harmonic wells forming equilateral triangles with side lengths 40 and 80 μm. In our approach, which is scalable to arbitrary two-dimensional lattices, we demonstrate individual control of the electronic and motional degrees of freedom, preparation of a fiducial initial state with ion motion close to the ground state, as well as a tuning of couplings between ions within experimental sequences. Our work paves the way towards a quantum simulator of two-dimensional systems designed at will.

## Abstract (web summary)

Analogue quantum simulators could provide insights into complex dynamics, but realizing them in a scalable way remains a technological challenge. Here, the authors demonstrate how two-dimensional arrays of three ions trapped in individually controlled wells could form a scalable platform for quantum simulation.

## Graphical abstract

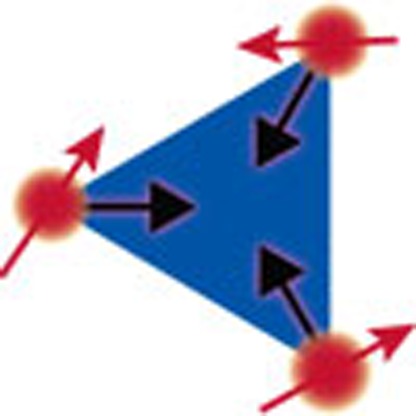

Richard Feynman was one of the first to recognize that quantum systems of sufficient complexity cannot be simulated on a conventional computer^1^. He proposed to use a quantum mechanical system instead. A universal quantum computer would be suitable, but practical implementations are a decade away at best. However, universality is not required to simulate specific quantum models. It is possible to custom-build an analogue quantum simulator (AQS) that allows for preparation of fiducial input states, faithful implementation of the model-specific dynamics and for access to the crucial observables. Simulations on such AQSs could impact a vast variety of research fields^2^, that is, physics^3^, chemistry^4^ and biology^5^, when studying dynamics that is out of reach for numerical simulation on conventional computers.

Many experimental platforms have been suggested to implement AQSs^6^,^7^,^8^,^9^. Different experimental systems provide certain advantages in addressing different physics. Results that are not conventionally tractable may be validated by comparing results of different AQSs simulating the same problem^10^,^11^. Over the last two decades, many promising proof-of-principle demonstrations have been made using photons^6^, superconductors^7^, atoms^8^ and trapped atomic ions^9^. Trapped ions in particular have seen steady progress from demonstrations with one or two ions^12^,^13^,^14^,^15^,^16^,^17^,^18^ to addressing aspects of quantum magnets^19^ with linear strings of 2–16 ions^13^,^20^ and self-ordered two-dimensional crystals containing more than 100 ions^21^. Ions are well suited to further propel the research since they provide long-range interaction and individual, fast controllability with high precision^22^.

Two-dimensional trap-arrays may offer advantages over trapping in a common potential, because they are naturally suited to implement tuneable couplings in more than one spatial dimension. Such couplings are, in most cases, at the heart of problems that are currently intractable by conventional numerics^10^,^23^. Our approach is based on surface-electrode structures^24^ originally developed for moving ion qubits through miniaturized and interconnected, linear traps as proposed in refs 25, 26. This approach is pursued successfully as a scalable architecture for quantum computer, see, for example, ref. 27. For AQSs, it is beneficial to have the trapped ion ensembles coupled all-to-all so they evolve as a whole. This is enabled by our array architecture with full control over each ion. Individual control allows us to maintain all advantages of single trapped ions while scaling the array in size and dimension^28^,^29^,^30^.

Optimized surface electrode geometries can be found for any periodic wallpaper group as well as quasi-periodic arrangements, as, for example, Penrose-tilings^29^. A first step, trapping of ions in two-dimensional arrays of surface traps, has been proposed^15^ and demonstrated^31^. Boosting the strength of interaction to a level comparable to current decoherence rates requires inter-ion distances *d* of a few tens of micrometres. Such distances have been realized in complementary work, where two ions have been trapped in individually controlled sites of a linear surface-electrode trap at *d* between 30 and 40 μm. The exchange of a single quantum of motion, as well as entangling spin–spin interactions have been demonstrated in this system^32^,^33^. The increase in coupling strength was achieved with a reduction of the ion-surface separation to order *d* and the concomitant increase in motional heating due to electrical noise. Recently, methods for reducing this heating by more than two orders of magnitude with either surface treatments^34^,^35^,^36^ or cold electrode surfaces^37^,^38^,^39^ have been devised.

Here, we demonstrate the precise tuning of all relevant parameters of a two-dimensional array of three ions trapped in individually controlled harmonic wells on the vertices of equilateral triangles with side lengths 80 and 40 μm. In the latter, Coulomb coupling rates^32^ approach current rates of decoherence. Dynamic control permits to reconfigure Coulomb and laser couplings at will within single experiments. We initialize fiducial quantum states by optical pumping, Doppler and resolved sideband cooling to near the motional ground state. Our results demonstrate important prerequisites for experimental quantum simulations of engineered two-dimensional systems.

## Results

### Trap arrays and control potentials

**Figure 1:**
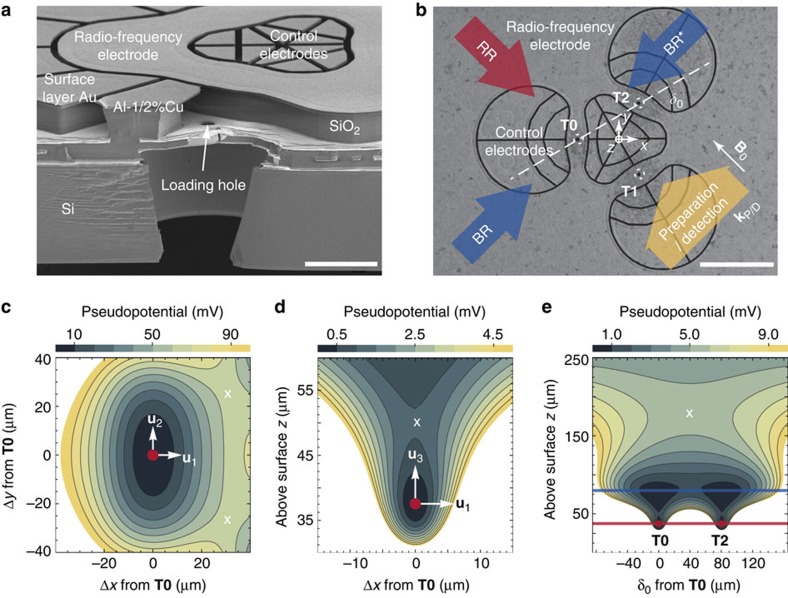
Surface-electrode ion trap featuring three individual traps. (**a**) Scanning electron microscope (SEM) image of a cleaved copy of our chip; white scale bar, 20 μm. It provides a cross-sectional view vertically through the trap chip (bottom half of image) and a top view of the horizontal, planar trap electrode surface (top half of image) of the 40 μm array. Buried electrode interconnects as well as the overhangs of electrodes that shield trapped ions from insulating surfaces are exposed in this view. A loading channel, vertically traversing the chip, collimates a neutral atom beam from an oven on the backside of the chip. (**b**) SEM top-view of the 80 μm array, dark lines indicate gaps between individual electrodes and dashed circles highlight the three trap sites at **T0**, **T1** and **T2** that lie 40 μm above the electrode plane (white scale bar, 80 μm); corresponding loading holes appear as dark spots. A vertical plane connecting **T0** and **T2** is shown as a dotted line and labelled with *δ*_0_. The single RF electrode extends beyond the image area and encloses 30 control electrodes grouped into four islands depicted in the central part of the image, enabling the control of individual trap sites. Laser beams (coloured arrows) are parallel to the chip surface and wave vectors **k**_P/D_ of preparation and detection beams are parallel to the magnetic quantization field **B**_0_ (white arrow). (**c**–**e**) The pseudopotential *φ*_ps_(*x,y,z*) of the 80 μm array in different planes is shown, trap sites marked by red dots, motional mode vectors 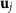 with *j*={1,2,3} at **T0** are represented by white arrows, and saddle points are illustrated by white crosses. In **e**, the heights of **T0**, **T2** and the ancillary trap are indicated by red and blue lines, respectively.

Our surface ion trap chip is fabricated in similar manner to that described in ref. 40 and consists of two equilateral triangular trap arrays with side length of ≃40 and ≃80 μm, respectively (Fig. 1a,b), both with a distance of ≃40 μm between the ions and the nearest electrode surface. The shapes of radio-frequency (RF) electrodes of the arrays are optimized by a linear-programming algorithm that yields electrode shapes with low fragmentation, and requires only a single RF-voltage source for operation^29^,^30^. To design different and even non-periodic arrays for dedicated trap distances, we can apply the same algorithm to yield globally optimal electrode shapes^29^. Resulting electrode shapes may look significantly different, but will have comparable complexity, spatial extent and the same number of control electrodes per trap site. Therefore, we expect that different arrays will not require different fabrication techniques (Methods). The two arrays are spaced by ≃5 mm on the chip, and only one of them is operated at a given time. Although we achieve similar results in both arrays, the following discussion is focussed on the 80 μm array.

Three-dimensional confinement of ^25^Mg^+^ ions is provided by a potential *φ*_RF_ oscillating at Ω_RF_ from a single RF electrode driven at Ω_RF_/(2*π*)=48.3 MHz with an approximate peak voltage *U*_RF_=20 V. Setting the origin of the coordinate system at the centre of the array and in the surface plane of the chip, the RF potential features three distinct trap sites at **T0**≃(−46,0,37) μm, 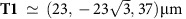, and 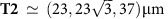. Owing to the electrode symmetry under rotations of ±2*π*/3 around the *z*-axis, it is often sufficient to consider **T0** only, as all our findings apply to **T1** and **T2** after an appropriate rotation. Further, the RF potential exhibits another trap site at ≃(0,0,81) μm (above the centre of the array); this ‘ancillary' trap is used for loading as well as for re-capturing ions that escaped from the other trap sites. We approximate the RF confinement at position **r** by a pseudopotential 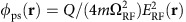, cp. ref. 41, where *Q* denotes the charge and *m* the mass of the ion, and *E*_RF_(**r**) is the field amplitude produced by the electrode. Calculations of trapping potentials are based on ref. 42 and utilizing the software package^43^. Equipotential lines of *φ*_ps_ are shown in Fig. 1c–e.

Near **T0** we can approximate *φ*_ps_ up to second order and diagonalize the local curvature matrix to find normal modes of motion described by their mode vectors **u**_1_, **u**_2_ and **u**_3_, which coincide (for the pure pseudopotential) with *x*, *y* and *z*; we use **u**_*j*_ with *j*={1,2,3} throughout our manuscript to describe the mode vectors of a single ion near **T0**. We find corresponding potential curvatures of *κ*_ps,1_≃3.0 × 10^8^ V m^−2^, *κ*_ps,2_≃5.9 × 10^7^ V m^−2^ and *κ*_ps,3_≃9.2 × 10^7^ V m^−2^, whereas mode frequencies can be inferred from these curvatures as 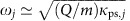, with *j*={1,2,3}: *ω*_1_/(2π)≃5.4 MHz, *ω*_2_/(2π)≃2.4 MHz and *ω*_3_/(2π)≃3.0 MHz. Further, the Mathieu parameters 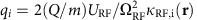, where *κ*_RF,i_(**r**) denotes the curvature of *φ*_RF_ along direction *i*={*x*,*y*,*z*}, at **T0** are: *q*_*x*_≃−0.32, *q*_*y*_≃0.14 and *q*_*z*_≃0.18.

To gain individual control of the trapping potential at each site, it is required to independently tune local potentials near **T0**, **T1** and **T2** (Methods), that is, to make use of designed local electric fields and curvatures. To achieve this, we apply sets of control voltages to 30 designated control electrodes (see Fig. 1 for details). In the following, a control voltage set is described by a unit vector 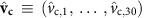, with corresponding dimensionless entries 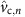 with *n*={1,…,30}, and result in a dimensionless control potential


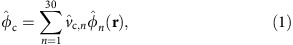


where 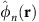 is the potential resulting when applying 1 V to the *n*-th electrode following a basis function method^44^,^45^. We scale 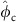 by varying a control voltage *U*_c_ and yielding a combined trapping potential


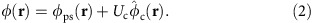


Bias voltages applied to the control electrodes are, in turn, fully described by 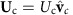.

To design a specific 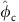, we consider the second order Taylor expansion for a point **r**_0_ and small displacements Δ**r**:


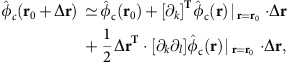


where 
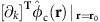
 is the local gradient and 
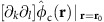
 is the traceless and symmetric matrix with indices *k* and *l*={*x*, *y*, *z*} that describes the local curvature; square brackets denote vectors/matrices, *∂* partial derivatives and the superscript **T** the transpose of a vector. We constrain local gradients in their three degrees of freedom (DoF) and local curvatures in their five DoF at **T0**, **T1** and **T2**, and solve the corresponding system of 24 linear equations to yield 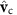. In principle, it would be sufficient to use 24 control electrodes, however, we consider all electrodes and use the extra DoF to minimize the modulus of the voltages we need to apply for a given effect.

In particular, we distinguish two categories of control potentials, denoted by 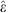 and 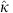, respectively: the first category is designed to provide finite gradients and zero curvatures at **T0**, with zero gradients and curvatures at **T1** and **T2**; for example, 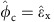 provides a gradient along 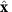 at **T0**. Control potentials of the second category are designed to provide zero gradients and only curvatures at **T0**, whereas we require related gradients and curvatures to be zero at **T1** and **T2**. For example, we design 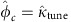, with the following non-zero constrains 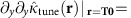 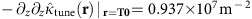 with corresponding *U*_c_=*U*_tune_. Linear combination of multiple control potentials enable us, for example, to locally compensate stray potentials up to second order, to independently control mode frequencies and orientations at each trap site, and, when implementing time-dependent control potentials, to apply directed and phase-controlled mode-frequency modulations or mode excitations.

### Optical setup and experimental procedures

We employ eight laser beams at wavelengths near 280 nm, from three distinct laser sources^46^, with wave vectors parallel to the *xy* plane (Fig. 1b) for preparation, manipulation and detection of electronic and motional states of ^25^Mg^+^ ions. Five distinct *σ*^+^-polarized beams (two for Doppler cooling, two for optical pumping and one for state detection) are superimposed, with wave vector **k**_P/D_ (preparation/detection) aligned with a static homogeneous magnetic quantization field **B**_**0**_≃4.65 mT (Fig. 1b). The beam waists (half width at 1/*e*^2^ intensity) are ≃150 μm in the *xy* plane and ≃30 μm in *z* direction, to ensure reasonably even illumination of all three trap sites, while avoiding excessive clipping of the beams on the trap chip. The two Doppler-cooling beams are detuned by Δ≃−Γ/2 and −10Γ (for initial Doppler cooling and state preparation by optical pumping) with respect to 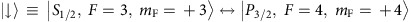 with a natural line width Γ/(2π)≃42 MHz. The state detection beam is resonant with this cycling transition and discriminates 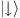 from 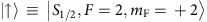, the pseudo-spin states 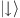 and 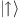 are separated by *ω*_0_/(2π)≃1,681.5 MHz. The resulting fluorescence light is collected with high numerical aperture lens onto either a photomultiplier tube or an electron-multiplying charge-coupled device camera. We prepare (and repump to) 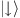 by two optical-pumping beams that couple 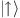 and 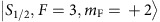 to states in 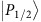 from where the electron decays back into the ground state manifold and population is accumulated in 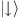. We can couple 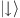 to 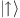 via two-photon stimulated-Raman transitions^25^,^47^,^48^, while we can switch between two different beam configurations labelled BR*+RR with 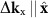 and BR+RR with 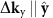. The beam waists are ≃30 μm in the *xy* plane and ≃30 μmm in *z* direction.

We load ions by isotope-selective photoionization from one of three atomic beams collimated by 4 μm loading holes located beneath each trap site (Fig. 1). We can also transfer ions from one site to any neighbouring site via the ancillary trap by applying suitable potentials to control electrodes and a metallic mesh (with high optical transmission) located ≃7 mm above the surface. Typically, experiments start with 2 ms of Doppler cooling, optionally followed by resolved sideband cooling, and 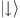 preparation via optical pumping. We use 30 channels of a 36-channel arbitrary waveform generator with 50 MHz update rate^49^ to provide static (persistent over many experiments) and dynamic (variable within single experiments) control potentials. Each experiment is completed by a pulse for pseudo-spin detection of duration ≃150 μs that yields ≃12 counts on average for an ion in 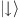 and ≃0.8 counts for an ion in 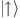. Specific experimental sequences are repeated 100–250 times.

Initially, we calibrate three (static) control potentials 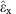, 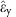 and 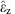 to compensate local stray fields^50^ with a single ion near **T0**, whereas we observe negligible effects on the local potentials near **T1** and **T2** (Methods). Rotated versions of these control potentials are used to compensate local stray fields near **T1** and **T2**. Near each site, we achieve residual stray field amplitudes ≤3 V m^−1^ in the *xy* plane and ≤900 V m^−1^ along *z*, currently limited by our methods for detection of micromotion.

With the stray fields approximately compensated, we characterize the trap near **T0** with a single ion (Methods). We find mode frequencies of *ω*_1_/(2*π*)≃5.3 MHz, *ω*_2_/(2*π*)≃2.6 MHz and *ω*_3_/(2*π*)≃4.1 MHz with frequency drifts of about 2*π* × 0.07 kHz (60 s)^−1^; mode frequencies and orientations are altered by local stray curvatures on our chip, in particular, **u**_1_ and **u**_3_ are rotated in the *xz* plane, while **u**_2_ remains predominantly aligned along *y*. We obtain heating rates for the modes **u**_1_ of 0.9(1) quanta ms^−1^, **u**_2_ of 2.2(1) quanta ms^−1^ and **u**_3_ of 4.0(3) quanta ms^−1^.

### Control of mode configurations at individual trap sites

**Figure 2:**
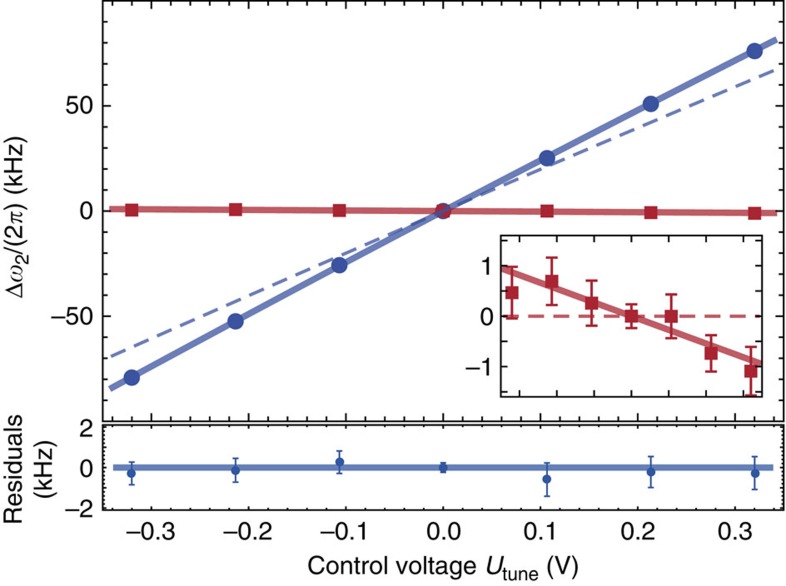
Individual control of mode frequencies. Frequency change Δ*ω*_2_/(2π) probed with a single ion near **T0** (blue dots) as a function of *U*_tune_. The intended control curvature 0.937 × 10^7^ m^−2^ (along the *y* direction) yields the blue/dashed line (cp. equation (4)), while a fit to the data (blue/solid line) returns a control curvature of 1.164(3) × 10^7^ m^−2^ (corresponding residuals shown in the bottom graph). The remaining change of the corresponding mode frequency of a single ion near **T2** (red squares) is shown in the inset, for the full range of *U*_tune_. A fit to these data (red/solid lines) results in a residual control curvature of −0.012(2) × 10^7^ m^−2^. Ideally, 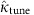 would create no additional curvature at **T2** (red/dashed line). We attribute the difference between designed and measured values to residual ion displacements from **T0** and **T2**. Each data point represents the average of 250 experiments with error bars (for some data smaller than symbols) denoting the s.e.m. Residual variations of experimental parameters, for example, changes of stray potentials, can result in day-to-day variations of measurement outcomes that require recalibration to remain within our stated statistical uncertainties.

The ability to control mode frequencies and orientations at each site with minimal effect on local trapping potentials at neighbouring sites is essential for the static and dynamical tuning of inter-ion Coulomb couplings. We experimentally demonstrate individual mode-frequency control using 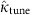. To this end we measure local mode frequencies with a single ion near **T0** or **T2** (Methods). Tuning of about ±2*π* × 80 kHz of *ω*_2_ near **T0** is shown in Fig. 2 as blue data points, accompanied by residual changes of about 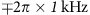 in the corresponding mode frequency near the neighbouring site **T2**, depicted by red data points. To infer local control curvatures, we describe the expected detuning Δ*ω*_2_ due to 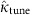 at **T0** (analogously at **T2**) by


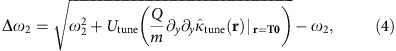


where we neglect a small misalignment of **u**_2_ from *y*. The prediction of equation (4) is shown as a blue/dashed line in Fig. 2. The blue/solid line results from a fit with a function of the form of equation (4) to the data yielding a control curvature of 1.164(3) × 10^7^ m^−2^. The inset magnifies the residual change in frequency near **T2**. Here, a fit (red/solid line) reveals a curvature of −0.012(2) × 10^7^ m^−2^. Residual ion displacements of Δ*z*=−2.95(3) μm from **T0** and Δ*z*=−2.9(4) μm from **T2**, respectively, suffice to explain deviations between experimentally determined and designed curvature values and are below our current limit of precision locating the ions in that direction. In future experiments, curvature measurements may be used to further reduce stray fields.

We also implement a dynamic *U*_tune_(*t*), to adiabatically tune *ω*_2_ near **T0** within single experiments: we prepare our initial state by Doppler cooling, followed by resolved sideband cooling of mode **u**_2_ to an average occupation number 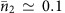 and optical pumping to 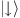. In a next step, we apply a first adiabatic ramp from *U*_tune,A_=0 V to *U*_tune,B_ between 0 and 2.3 V (corresponding to a measured frequency difference Δ*ω*_2_/(2*π*)≃430 kHz) within *t*_ramp_=7.5 to 120 μs and, subsequently, couple 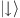 and 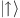 to mode **u**_2_ with pulses of BR+RR tuned to sideband transitions that either add or subtract a single quantum of motion. If the ion is in the motional ground state, no quantum can be subtracted and the spin state remains unchanged when applying the motion subtracting sideband pulse. The motion-adding sideband can always be driven, and comparing the spin-flip probability of the two sidebands allows us to determine the average occupation of the dynamically tuned mode^48^. We find that the average occupation numbers are independent of the duration of the ramp and equal to those obtained by remaining in a static potential for *t*_ramp_, that is, the motion is unaffected by the dynamic tuning.

**Figure 3:**
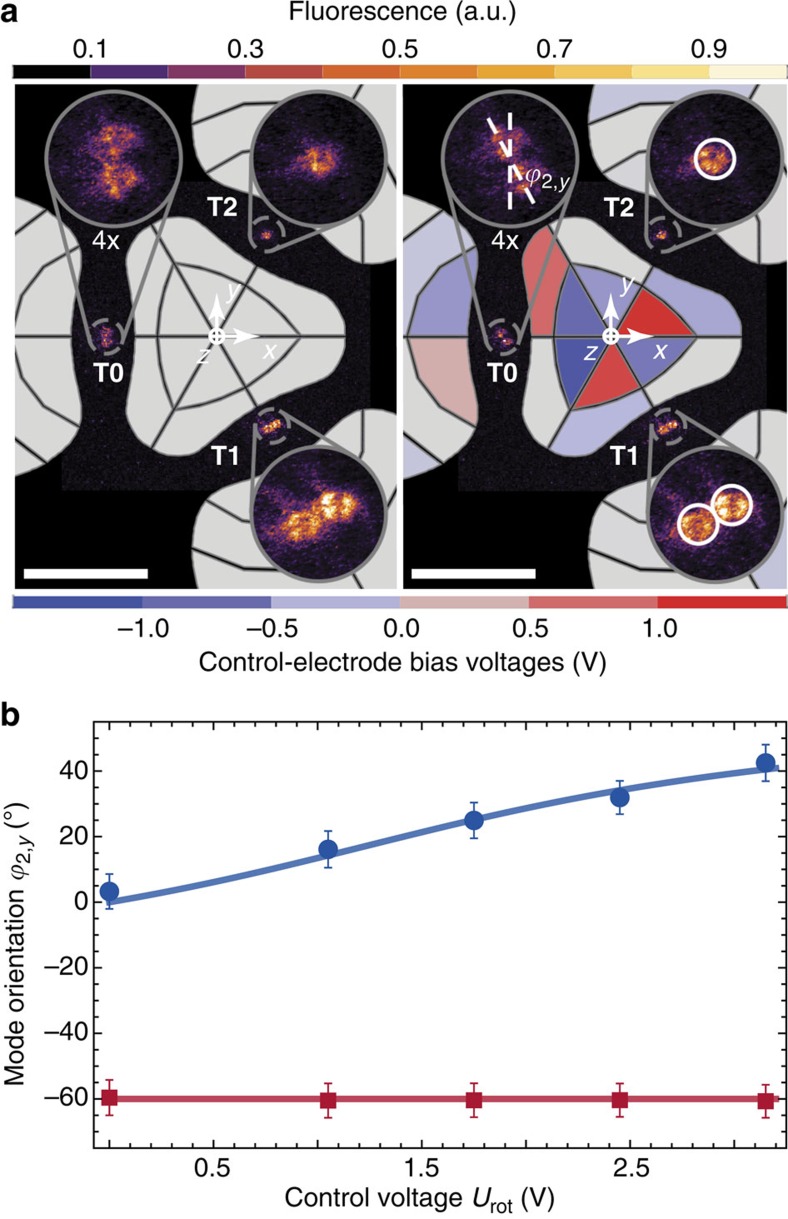
Individual control of mode orientations. (**a**) Electron-multiplying charge-coupled device images of pairs of ions near **T0** and **T1**, and a single ion near **T2**; white scale bars are 50 μm and blow ups of each site are magnified by four. Schematics of control electrodes are coloured according to their bias voltage ***U***_rot_. Ion pairs align along **u**_2_, the lowest-frequency mode. The left image captures ion positions for *U*_rot_=0 V and, here, *ω*_2_/(2*π*)=1.9(2) MHz near **T0**. The right image illustrates the rotation effect for *U*_rot_=2.45 V: Mode **u**_2_ near **T0** with *ω*_2_/(2*π*)=1.8(2) MHz is rotated by *ϕ*_2,y_=31(5)°, whereas ion positions near **T1** and **T2** remain unchanged; white circles indicate initial ion positions (for *U*_rot_=0). (**b**) Mode **u**_2_ orientation in the *xy* plane for **T0** (blue dots) and **T1** (red squares), described by *ϕ*_2,*y*_, derived from a total of 14 images as a function of *U*_rot_; error bars denote our systematic uncertainty. The data are in good agreement with our predictions of the effect of 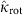 (solid lines).

We rotate mode orientations near **T0** in the *xy* plane with a control-potential 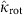, while setting additional constraints to keep gradients and curvatures of the local trapping potential constant at **T1** and **T2** (Methods). We determine the rotation of mode orientations from electron-multiplying charge-coupled device images of two ions near **T0** that align along **u**_2_ (axis of weakest confinement). Simultaneously, we trap one or two ions near **T1** and **T2** to monitor residual changes in ion positions and mode orientations (and frequencies) because of unwanted local gradients and curvatures of 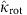. We take 14 images for five different 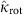 values, while constantly Doppler cooling all ions and exciting fluorescence. Figure 3a shows two images for *U*_rot_=0 V (left) and *U*_rot_=2.45 V (right). Schematics of control electrodes are overlaid to the images and coloured to indicate their bias voltages *U*_rot_. Ion positions (in the *xy* plane) are obtained with an uncertainty of ±0.5 μm, yielding uncertainties for inferred angles *ϕ*_2,*y*_ of ±5°. Here, *ϕ*_2,*y*_ denotes the angle between local mode **u**_2_ and *y*. Figure 3b shows measured *ϕ*_2,*y*_ for ions near **T0** (blue dots) and **T1** (red squares) and compares them with our theoretical expectation (solid lines), further described in the Methods. We tune *ϕ*_2,*y*_ between 0° and 45° near **T0**, enabling us to set arbitrary mode orientations in the *xy* plane, whereas ion positions (mode orientations) near **T1** and **T2** remain constant within ±0.5 μm (better than ±5°) in the *xy* plane.

A complementary way of characterizing mode orientations and frequencies, now with respect to **Δk**_*x*_ and/or **Δk**_*y*_ is to analyse the probability of finding 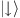 after applying 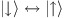 (carrier) or motional sideband couplings for variable duration. If all modes of a single ion are prepared in their motional ground state, the ratio of Rabi frequencies of carrier and sideband couplings is given by the Lamb-Dicke parameter^48^, which is for **u**_1_ and **Δk**_*x*_:


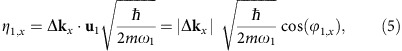


where *ϕ*_1,*x*_ is the angle between **u**_1_ and **Δk**_*x*_. The differences of carrier and sideband transition frequencies reveal the mode frequencies, whereas ratios of sideband and carrier Rabi-frequencies determine Lamb-Dicke parameters and allow for finding the orientation of modes.

**Figure 4:**
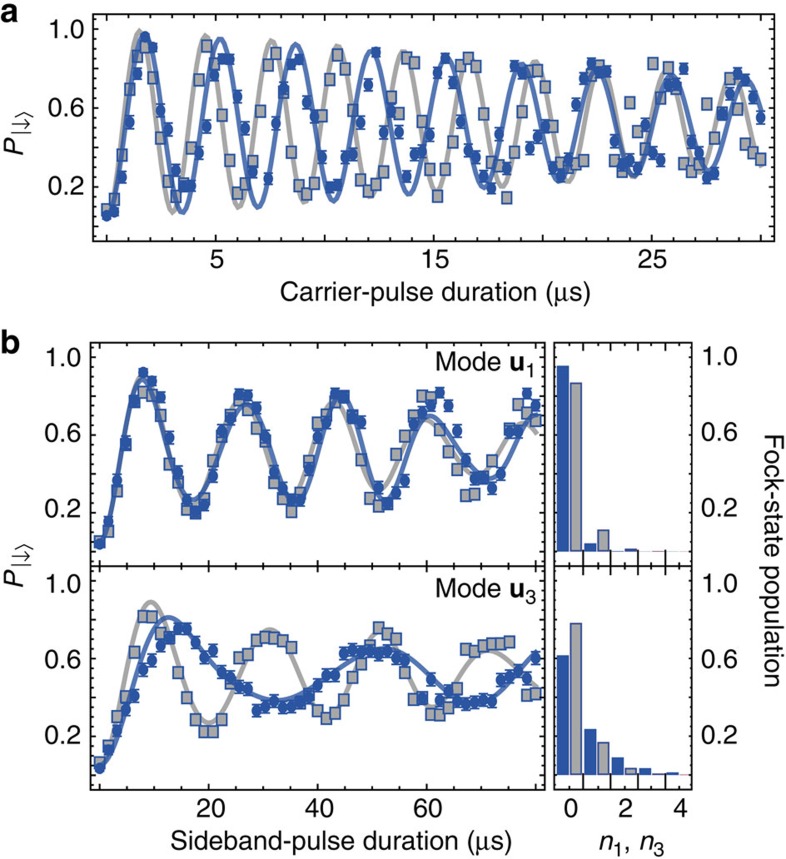
Measuring mode orientations via laser couplings. Probability of 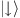 after applying carrier and sideband couplings via **Δk**_*x*_ with a single ion near **T0**. Mode orientations are set with static 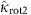 (rotations in the *xz* plane) for *U*_rot2_=1.62 V (blue dots) and −2.43 V (grey squares). (**a**) Shows the carrier transitions, 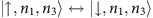, whereas **b** represents the **u**_1_-sideband transitions, 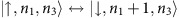, and the **u**_3_-sideband transitions, 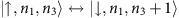. From combined model fits to all transitions (for each 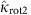), we find angles *ϕ*_1,*x*_=24.7(2)° for *U*_rot2_=−1.62 V (blue lines) and 36.1(2)° for *U*_rot2_=−2.43 V (grey lines) of mode **u**_1_ relative to **Δk**_*x*_. Histograms in **b** display derived Fock-state populations with thermal average occupation numbers between ≃0.05 and ≃0.6. Each data point is the average of 250 experiments and error bars (for some data smaller than symbols) denote the s.e.m. Residual variations of experimental parameters, for example, changes of stray potentials, can result in day-to-day variations of measurement outcomes that require recalibration to remain within our stated statistical uncertainties.

We use a single ion near **T0** to determine the orientations and frequencies of two modes relative to **Δk**_*x*_. We apply another control potential 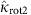, designed to rotate **u**_1_ and **u**_3_ in the *xz* plane near **T0**, and implement carrier and sideband couplings to both modes with **Δk**_*x*_ after resolved sideband cooling and initializing 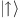. In Fig. 4, the probability of 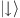 is shown for different pulse durations of carrier couplings (top) and sideband couplings to mode **u**_1_ (middle) and **u**_3_ (bottom). Data points for *U*_rot2_=−1.62 V are shown as blue rectangles and for −2.43 V as grey rectangles. We fit each data set to a theoretical model (blue and grey lines) to extract the angles^51^ and distributions of Fock-state populations of each mode (shown as histograms): we find *ϕ*_1,*x*_=24.7(2)° for *U*_rot2_=−1.62 V and *ϕ*_1,*x*_=36.1(2)° for *U*_rot2_=−2.43 V, whereas average occupation numbers range between ≃0.05 and ≃0.6. Adding measurements along **Δk**_*y*_ and taking into account that the normal modes have to be mutually orthogonal would allow to fully reconstruct all mode orientations. With resolved sideband cooling on all three modes, we can prepare a well-defined state of all motional DoF.

## Discussion

We characterized two trap arrays that confine ions on the vertices of equilateral triangles with side lengths 80 and 40 μm. We developed systematic approaches to individually tune and calibrate control potentials in the vicinity of each trap site of the 80-μm array, by applying bias potentials to 30 control electrodes. With suitably designed control potentials, we demonstrated precise individual control of mode frequencies and orientations. By utilizing a multi-channel arbitrary waveform generator, we also dynamically changed control potentials within single experimental sequences without adverse effects on spin or motional states. Further, we devised a method to fully determine all mode orientations (and frequencies) based on the analysis of carrier and sideband couplings. Measured heating rates are currently comparable to the expected inter-ion Coulomb coupling rate of Ω_ex_/(2*π*)≃1 kHz for ^25^Mg^+^ ions in the 40-μm array at mode frequencies of ≃2*π* × 2 MHz (ref. 32). This coupling rate sets a fundamental time scale for effective spin–spin couplings^33^. To observe coherent spin–spin couplings, ambient heating needs to be reduced. Decreases in heating rates of up to two orders of magnitude would leave Ω_ex_ considerably higher than competing decoherence rates and allow for coherent implementation of fairly complex spin–spin couplings. Such heating rate reductions have been achieved in other surface traps by treatments of the electrode structure^34^,^35^,^36^ and/or cryogenic cooling of the electrodes^37^,^38^,^39^. The couplings in question have been observed in one dimension in a cryogenic system^32^,^33^.

**Figure 5:**
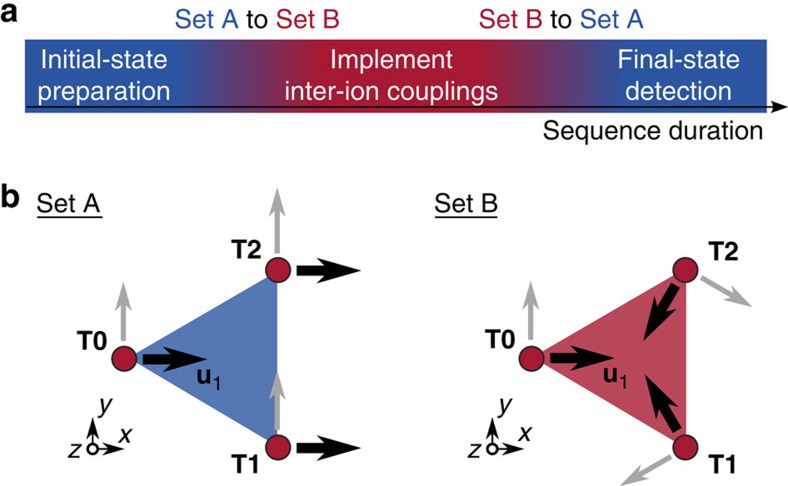
Generic experimental sequence for tuneable inter-ion couplings. (**a**) Time-line of an experiment starting with the initialization of an fiducial quantum state of all ions in the array, followed by an adiabatic (or diabatic) ramp of control potentials between sets A and B that reconfigure the normal-mode structure from the setup, see **b**. Then, appropriate laser fields implement inter-ion couplings required for the AQS. The simulation completes with ramping the control potentials back to set A, where the individual spin states can be detected. (**b**) Examples for configurations of motional DoF are illustrated by the arrows that show the orientation of **u**_1_ at the three trap sites (red dots). Set A may be applied when globally and/or locally preparing and detecting spin-motional states, whereas set B can establish specific inter-ion Coulomb couplings to mediate, for example, effective spin–spin couplings for AQS, cp. refs 33, 55.

Currently, we can compensate stray fields, set up normal mode frequencies and directions for all three ions and initialize them for a two-dimensional AQS, that is, prepare a fiducial initial quantum state for ions at each trap site. A complete AQS may use the sequence presented in Fig. 5. A dynamic ramp adiabatically transforms the system between two control sets, labelled as A and B, that realize specific mode frequencies and orientations at each site. Set A may serve to globally initialize spin-motional states of ions, potentially with more than one ion at each site, that could be the ground state of a simple initial Hamiltonian. At all sites, mode frequencies and orientations need to be suitable (bottom left of Fig. 5) to enable global resolved sideband cooling, ideally preparing ground states for all motional modes. A first ramp to set B combined with appropriate laser fields may be used to adiabatically or diabatically realize a different Hamiltonian, for example, by turning on complex spin–spin couplings. Mode frequencies and orientations are tuned such that the Coulomb interactions between ions can mediate effective spin–spin couplings, for example, all mode vectors **u**_1_ are rotated to point to the centre of the triangle (bottom right of Fig. 5). During the application of such interactions, the ground state of the uncoupled system can evolve into the highly entangled ground state of a complex coupled system. In contrast, diabatic ramping to set B will quench the original ground state and the coupled system will evolve into an excited state that is not an eigenstate. After a final adiabatic or diabatic ramp back to set A, we can use global (or local) laser beams to read out the final spin states at each site.

In this way, our arrays may become an arbitrarily configurable and dynamically reprogrammable simulator for complex quantum dynamics. It may enable, for example, the observation of photon-assisted tunnelling, as required for experimental simulations of synthetic gauge fields^52^,^53^ or other interesting properties of finite quantum systems, such as thermalization, when including the motional DoF^54^. Concentrating on spin–spin interactions, the complex entangled ground states of spin frustration can be studied in the versatile testbed provided by arrays of individually trapped and controlled ions^30^,^55^. Arrays with a larger number of trap sites could realize a level of complexity impossible to simulate on conventional computers^56^,^57^.

## Methods

### Design of arrays used in the experiments

The design of arrays used in the expeiments is based on the methods described in ref. 29. In particular, we use the Mathematica package for surface atom and ion traps^43^ to globally optimize the RF electrode shape for maximal curvature with a given amplitude of the RF drive, whereas producing smooth continuous electrode shapes that require a single RF drive to operate the array. We specify the desired trap site positions as well as the ratio and orientation of normal-mode frequencies as a fixed input to the optimization algorithm for the pseudopotential, that is, we define that the high-frequency mode (for all three sites) lies within the *xy* plane and points towards the virtual centre of the array. Resulting electrode regions held to ground are subdivided into separated control electrodes that provide complete and independent control over the eight DoF at each site.

### Array scaling for future realisations

**Figure 6:**
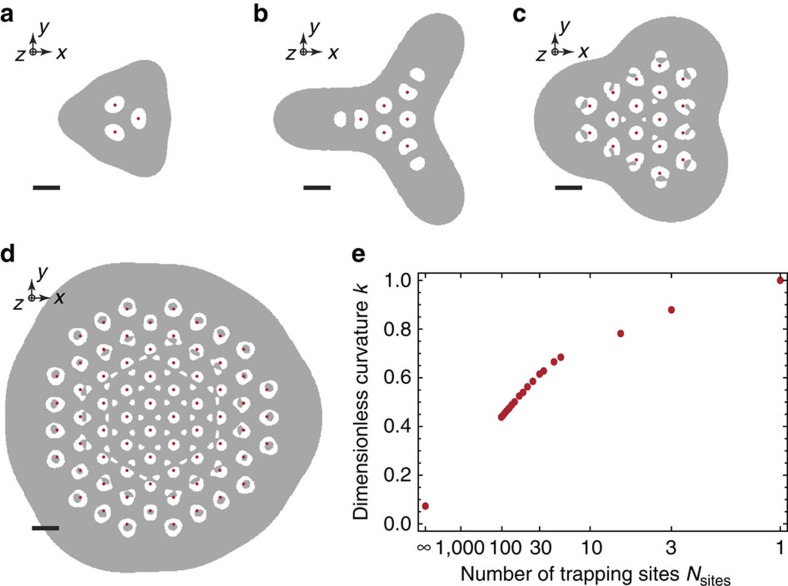
Scale-up the number of sites in triangular arrays. (**a**–**d**) Optimized surface-electrode shapes for 3, 6, 18 and 69 trapping sites, where we assume *h/d*=1/2 (as for our 80 μm array); black scale bars have fixed length *d*. The high-frequency mode is aligned orthogonally to the *xy* plane in each trapping site, to permit comparability of the different geometries. RF and ground electrodes are coloured in grey and white, respectively, and individual trap sites are marked with red dots. In a functional array, we will have to further subdivide the ground electrodes to enable individual control at each site, as demonstrated in our work with three sites. (**e**) Optimized dimensionless curvature *κ* as a function of the number of trapping sites in the triangular arrays, quantifying the strength of the individual traps. For a single site *κ*=1.0 and for an infinite triangular lattice *κ*≃0.07. In experimental realizations, we can compensate this decrease in trap curvature by adjusting *U*_RF_ and Ω_RF_, or by reducing *h*.

To ensure that our approach can be scaled to more than three trapping sites, we compare designs of arrays containing different numbers of sites, *N*_sites_, that are optimized by the algorithm described in ref. 29. Here, we assume a fixed ratio of *h*/*d*=1/2, where *h* denotes the distance of the sites to the nearest electrode surface and *d* is the inter-site distance. Further, we specify for all arrays that the high-frequency mode is aligned orthogonally to the *xy* plane at each site, in contrast to our demonstrated arrays (see Fig. 1 for details). This unique mode configuration permits a fair comparison of geometries with increasing *N*_sites_. To illustrate the optimal electrode shapes, we present four examples of triangular arrays with *N*_sites_={3,6,18,69} in Fig. 6a–d. To enable the same level of individual control as demonstrated for both of our three-site arrays, we would have to subdivide the optimized ground electrodes into ≥8 × *N*_sites_ control electrodes. We find that the inner areas converge to fairly regular electrode shapes for larger *N*_sites_, whereas electrodes closer to the border are deformed to compensate for edge effects (see Fig. 6d for details). However, the spatial extent and complexity of all electrodes remains comparable to the arrays used in our experiments and, thus, fabrication of these larger arrays can be accomplished by scaling the applied techniques (see below).

To quantify the geometric strength of individual trap sites independently of *m*, *U*_RF_, Ω_RF_ and *h*, we consider the dimensionless curvature *κ* of the pseudopotential that we normalize to the highest possible curvature for a single site^29^. We show optimized *κ* for arrays with *N*_sites_ between 1 and 102, as well as, the value for *N*_sites_=∞ in Fig. 6e; a fully controlled array with *N*_sites_=102 should be sufficient to study quantum many-body dynamics that are virtually impossible to simulate on a conventional computer. We find that *k* for *N*_sites_=102 is reduced by about a factor of two compared with *κ*≃0.87 for *N*_sites_=3, whereas *κ*≃0.07 for *N*_sites_=∞; see ref. 29 for a detailed discussion of infinite arrays. The decrease in trap curvature can be compensated in experiments by adjusting *U*_RF_ and Ω_RF_ correspondingly, or by reducing *h*. Further, we estimate that trapping depths remain on the same order of magnitude for increasing *N*_sites_ compared with our demonstrated arrays (cp. Fig. 1d). For an infinite array it has been shown that depths of a few mV are achievable^30^. Note, that in surface-electrode traps the trapping potential is less deep along *z* than in the *xy* plane, and ion-escape points (closest and lowest saddle point of the pseudopotential) typically lie above each site. In experiments, we may apply a constant bias potential to the control electrodes, surrounding ground planes, and the mesh (cover plane) to increase the depth along *z* to a level where trapping is routinely achieved, while reducing the depth in the *xy* plane^30^. With such measures in place, we are fairly confident that ions created by photoionization from a hot atomic beam can be loaded and cooled into the local minima of larger arrays.

### Architecture of our trap chip

The 10 × 10 mm^2^ Si substrate of our trap chip is bonded onto a 33 × 33 mm^2^ ceramic pin grid array (CPGA); the electrodes of the trap arrays are wire-bonded with aluminium wires to the pins of the CPGA, with independent pins for the RF electrodes of the two arrays. The trap chip contains four aluminum-1/2% copper metal layers, that are electrically connected by tungsten vertical interconnects thereby allowing ‘islanded' control electrodes in the top electrode layer (Fig. 1). The buried electrical leads are isolated by intermediate SiO_2_ layers, nominally 2 μm thick, while the surface layer is spaced by 10 μm from the buried layers. All electrodes are mutually separated by nominally 1.2–1.4 μm gaps and a 50-nm gold layer is evaporated on the top surfaces in a final fabrication step. The trap chip fabrication is substantially the same as that described in the Supplement to ref. 40. Each control electrode is connected to ground by 820 pF capacitors located on the CPGA to minimize potential changes due to capacitive coupling to the RF electrodes.

### Compensation of stray potentials at each site

For compensation of local stray fields in the *xy* plane, we vary the strength of individual control potentials 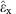 and 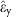 and find corresponding coefficient settings where we obtain a maximal Rabi rate of the detection transition and/or minimal Rabi rates of micromotion-sideband transitions probed with **Δk**_x_ and **Δk**_y_; resulting in residual stray-field amplitudes of ≤3 V m^−1^. For compensation along *z*, we vary the strength of individual 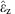 to minimize a change in ion position due to a modulation of *U*_RF_. The depth of field of our imaging optics aids to detect changes in *z*-position via blurring of images of single ions trapped at each site, within an uncertainty of about ±5 μm. This corresponds to residual stray-field amplitudes of ≃900 V m^−1^ for typical trapping parameters.

### Mode frequency and heating rate measurements

To measure mode frequencies, we Doppler-cool the ion and pump to 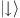. Then, we apply a motional excitation pulse with fixed duration *t*_exc_=100 μs to a single control electrode. The pulse produces an electric field oscillating at a frequency *ω*_exc_ that excites the motion, if *ω*_exc_ is resonant with a mode frequency, and we can detect mode amplitudes of >100 nm along **k**_D_ via the Doppler effect. In the experiments, we vary *ω*_exc_ and obtain resonant excitations at *ω*_*j*_ with *j*={1,2,3}. By repeating measurements, we record ≃50 consecutive frequency values for each mode frequency over the course of Δ*t*≃1 h with a single ion near **T0**. The results are consistent with linear changes in frequencies, with rates Δ*ω*_1_/Δ*t*=−2*π* × 0.090(3) kHz (60 s)^−1^, Δ*ω*_2_/Δ*t*=−2*π* × 0.064(1)  kHz (60 s)^−1^ and Δ*ω*_3_/Δ*t*=−2*π* × 0.063(5) kHz (60 s)^−1^.

For the heating rate measurements, we add multiple resolved-sideband cooling pulses after Doppler cooling to our sequence and determine mode temperatures from the sideband ratios for several different delay times^58^. In our experiments, we either use **Δk**_x_ to iteratively address **u**_1_ and **u**_3_ or **Δk**_y_ to address only **u**_**2**_. For this, we prepare similar mode orientations as presented in Fig. 4, find initial mode temperatures after cooling to 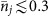, and obtain corresponding heating rates.

### Potentials for individual control

As a representative example for designing control potentials, we discuss 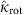 that serves to rotate the normal modes in the *xy* plane. At position **T0**, the constraints are:


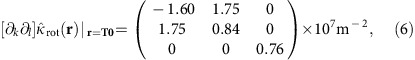


for *k* and *l*={*x*,*y*,*z*}, while local gradients at all three trap sites and local curvatures at **T1** and **T2** are required to be zero. We add diagonal elements in 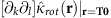 to reduce changes of the **u**_2_ frequency during variation of 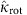 around our initial mode configurations. The mode configurations in the real array deviate from those derived from the *φ*_ps_ due to additional curvatures near each trap site generated by stray potentials on our chip. Ideally, we would design control potentials for mode rotations such that all frequencies stay fixed. This is only possible if we explicitly know the initial mode configuration. In addition, we keep mode vectors tilted away from *z* to sufficiently Doppler cool all modes during state initialization. Similarly, we design 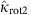 to rotate modes in the *xz* plane.

### Model for varying mode orientations

To model the rotation angle *ϕ*_2,*y*_ of **u**_2_ near **T0** as a function of 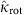, we consider the final trapping curvature at **T0** (analogously for neighbouring sites):


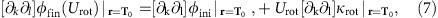


where *φ*_ini_(**r**) represents the initial potential, that is, the sum of the pseudopotential, stray potential and additional control potentials (used for stray field compensation). The local curvatures (mode frequencies and vectors) of *φ*_ini_(**r**) near **T0** are estimated from calibration experiments. For simplicity, we reduce equation (7) to two dimensions (in the *xy* plane) and find corresponding eigenvectors and eigenvalues for *U*_rot_ between 0.0 and 3.0 V. We obtain angles *ϕ*_2,*y*_(*U*_rot_) of the eigenvector **u**_2_ and we show resulting values as an interpolated solid line in Fig. 3b.

Similarly, we model the effect of 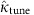 on *ω*_2_. We assume that for *U*_tune_=0, the corresponding mode vector **u**_2_ is aligned parallel to *y*. This is the case for pure RF confinement (cp. Fig. 1c) and sufficiently small stray curvatures. We design 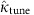 to tune the curvature along *y*, and the curvature as a function of *U*_tune_ (along this axis) is described by: 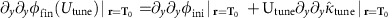. Finally, we insert this into 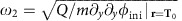 to find equation (4).

### Data availability

The data that support the findings of this study are available from the corresponding author upon request.

## Additional information

**How to cite this article:** Mielenz, M. *et al.* Arrays of individually controlled ions suitable for two-dimensional quantum simulations. *Nat. Commun.* 7:11839 doi: 10.1038/ncomms11839 (2016).
